# The effect of self-concealment on post-traumatic stress symptoms in breast cancer patients: The mediating role of experiential avoidance

**DOI:** 10.1371/journal.pone.0342971

**Published:** 2026-02-19

**Authors:** Fan Xu, Shaoju Xie, Qiao Li, Xiaoli Zhong, Jiquan Zhang

**Affiliations:** 1 Oncology Department, Deyang People’s Hospital, Deyang, Sichuan Province, China; 2 Nursing Department, Deyang People’s Hospital, Deyang, Sichuan Province, China; 3 Nephrology Department, Deyang People’s Hospital, Deyang, Sichuan Province, China; Sichuan University, CHINA

## Abstract

**Background:**

Breast cancer is a major global health issue. It brings death threats or serious physical injuries to patients, and is a traumatic event that can seriously affect their mental health and lead to post-traumatic stress, which can lead to serious physical, psychological, cognitive, and social dysfunction, and even increase the risk of suicide, and impose a heavy burden on patients and their families.

**Aim:**

To investigate the mediating effect of experiential avoidance between self-concealment and post-traumatic stress symptoms in breast cancer patients.

**Methods:**

This study used a cross-sectional survey design. From 15/08/2021 to 31/12/2021, breast cancer patients were recruited as study subjects in the oncology department of a tertiary hospital in Deyang City. Data were collected through the following tools: the general information questionnaire, the Impact of Event Scale-Revised, the Self-Concealment Scale, and the Acceptance and Action Questionnaire-Second Edition.

**Results:**

257 breast cancer patients eventually completed the study, all females. Descriptive results showed that breast cancer patients had self-concealment scores (24.75 ± 7.34), experiential avoidance scores (18.48 ± 5.44), and post-traumatic stress symptoms scores (32.29 ± 14.14). Pearson correlation analyses showed that self-concealment was positively correlated with experiential avoidance and traumatic stress response (r = 0.343, 0.467, both P < 0.01); experiential avoidance was positively correlated with traumatic stress response (r = 0.534, P < 0.01). Mediation effect analyses showed that the total effect of self-concealment on post-traumatic stress symptoms was 0.453, with a direct effect path coefficient of 0.310, and the mediation effect path coefficient of experiential avoidance between self-concealment and post-traumatic stress symptoms was 0.142 (95% CI: 0.074 to 0.223), accounting for 31.35% of the total effect.

**Conclusion:**

Experiential avoidance in breast cancer patients mediates the relationship between self-concealment and posttraumatic stress symptoms. and could guide healthcare professionals in developing tailored interventions to improve the mental health of patients.

## Introduction

According to the latest data from the International Agency for Research on Cancer (IARC) in 2022, breast cancer has a worldwide incidence of 2.29 million cases, making it the most common cancer globally [1]. In China, there are 420,000 new cases, ranking it as the sixth most common cancer overall and the most common cancer among women, significantly impacting women’s health in the country. With the incidence of breast cancer rising annually and the 5-year survival rate increasing, the population of breast cancer patients has become substantial. Cancer poses a threat of death or serious physical harm and is a traumatic event that can significantly affect patients’ mental health, leading to psychological reactions such as fear, avoidance, and hypervigilance. Currently, surgery, radiotherapy, and chemotherapy remain the standard treatments for breast cancer, and all these treatments can cause changes in patients’ body image, such as loss of secondary sex characteristics, hair loss, and pigmentation. Along with the distress of disease symptoms and the pressure from family and society, patients often experience mood swings, changes in temperament, and feelings of self-denial and isolation, leading to difficulties in social functioning and further exacerbating their psychological trauma.

Post-traumatic stress symptoms (PTSS) are delayed-onset and long-lasting stress responses following exposure to an extremely threatening or catastrophic event [2]. PTSS include three main features: intrusiveness, avoidance, and hypervigilance. Intrusiveness involves the recurrent reliving of the traumatic event through intrusive memories and nightmares; avoidance involves steering clear of activities or situations related to the traumatic event; and hypervigilance is a heightened state of alertness to potential threats in the environment. The Diagnostic and Statistical Manual of Mental Disorders (Fifth Edition) lists having a life-threatening illness as a potentially traumatic event [3].Developing breast cancer is a major traumatic event for patients. Studies have reported that breast cancer patients experience varying degrees of PTSS, with the prevalence of post-traumatic stress disorder (PTSD) reaching up to 24.1% [4]. These symptoms can lead to significant physical, psychological, cognitive, and social dysfunction [5], and even increase the risk of suicide, placing a heavy burden on patients and their families [6,7]. Research has noted a considerable delay between the onset of PTSS and the initiation of treatment [8]. Therefore, early identification and detection of these symptoms, along with timely implementation of both short-term and long-term psychological interventions, are crucial to help patients alleviate the decline in decision-making, communication, and socialization caused by psychological symptoms. Given the above, this study aims to investigate the psychological risk factors for PTSS in breast cancer patients and their potential mediating mechanisms.

Self-concealment is the tendency of individuals to actively hide distressing or negative personal information from others [9]. Any factor that causes an individual to worry about daily life and social interactions, such as serious illness, traumatic experiences, or negative thoughts, may motivate self-concealment. The Self-Concealment Working Mode states that self-concealment is an independent inhibitory personality trait associated with social anxiety. This can affect an individual’s psychological well-being and contribute to psychological disorders such as PTSS through secret-keeping behaviors and maladaptive emotion regulation strategies [10]. In breast cancer patients, patients may tend to conceal treatment side effects (e.g., hair loss, fatigue) or postoperative body image changes (e.g., missing breasts) due to stigma and family burdens, adopt nonadaptive strategies such as catastrophic thinking, rumination contemplation, and hypervigilance, which are behaviors that cause patients to be overly pessimistic about the disease’s prognosis, to repeatedly reminisce about traumatic experiences, and to become overly sensitive to small changes in their bodies, thereby exacerbating intrusive symptoms and significantly increasing the risk of PTSS [11,12].Studies have shown that self-concealment is a personality trait risk factor for PTSS [13,14]. It is positively correlated with physical symptoms, anxiety, depression, psychological distress, and other health problems. The higher the tendency for self-concealment, the more severe the individual’s physical and mental health problems. Additionally, patients who tend to self-conceal are often unwilling to seek professional help for psychological problems, leading to further exacerbation of their issues. A study of firefighters noted that the more pronounced an individual’s motivation for self-concealment, the greater the severity of PTSS [15].Conversely, recounting trauma aloud (the opposite of self-concealment) can help process traumatic memories, reduce distress and avoidance caused by the traumatic event, and be effective in treating PTSD [16].

Experiential avoidance is a potentially maladaptive self-regulatory tendency where individuals try to avoid negative thoughts, emotions, memories, bodily sensations, and the events or stimuli that produce these internal experiences [17]. Previous research has shown that self-concealment as a personality trait positively predicts experiential avoidance [15]. As a common emotion regulation strategy, experiential avoidance is one of the six core elements of psychological rigidity. Attempts to avoid traumatic events can worsen the psychological trauma associated with them and may lead to mental health problems such as PTSS [18]. According to Dual Representation Theory, trauma memories are divided into Verbalizable Accessible Memories (VAMs) and Situationally Accessible Memories (SAMs). Experiential avoidance can hinder the integration of VAMs and SAMs, leaving trauma memories fragmented and contributing to ongoing post - traumatic stress symptoms (PTSS). For breast cancer patients, traumatic experiences like surgical pain, fear of cancer, and changes in body image during diagnosis and treatment can create both VAMs and SAMs. VAMs are reflected in patients’ verbal descriptions of their illness and treatment, while SAMs are shown in emotional responses like fear and anxiety triggered by specific sounds, smells, or bodily pains related to treatment settings. When breast – cancer patients exhibit experiential avoidance, VAMs are temporarily kept out of consciousness. Consequently, sensory information and emotional experiences in SAMs can’t connect with verbal information and episodic memories in VAMs. In the long run, un-integrated SAMs are easily activated by future situations, causing flashbacks and nightmares. As these PTSS lack adaptive processing, they become hard to alleviate and may even worsen [19]. Previous studies have identified experiential avoidance as a psychological risk factor for PTSS and a positive predictor of post-traumatic stress [20,21]. Higher levels of PTSS can also increase an individual’s experiential avoidance [22]. Emotion regulation strategies, such as experiential avoidance, can mediate the effects of personality traits like self-concealment on PTSS [23].

Accordingly, this study proposes the following hypotheses:

H1: Self-concealment in breast cancer patients positively predicts PTSS.

H2. Self-concealment in breast cancer patients positively predicts experiential avoidance.

H3. Experiential avoidance in breast cancer patients positively predicts PTSS.

H4. Experiential avoidance in breast cancer patients mediates the relationship between self-concealment and PTSS.

**Fig 1:**
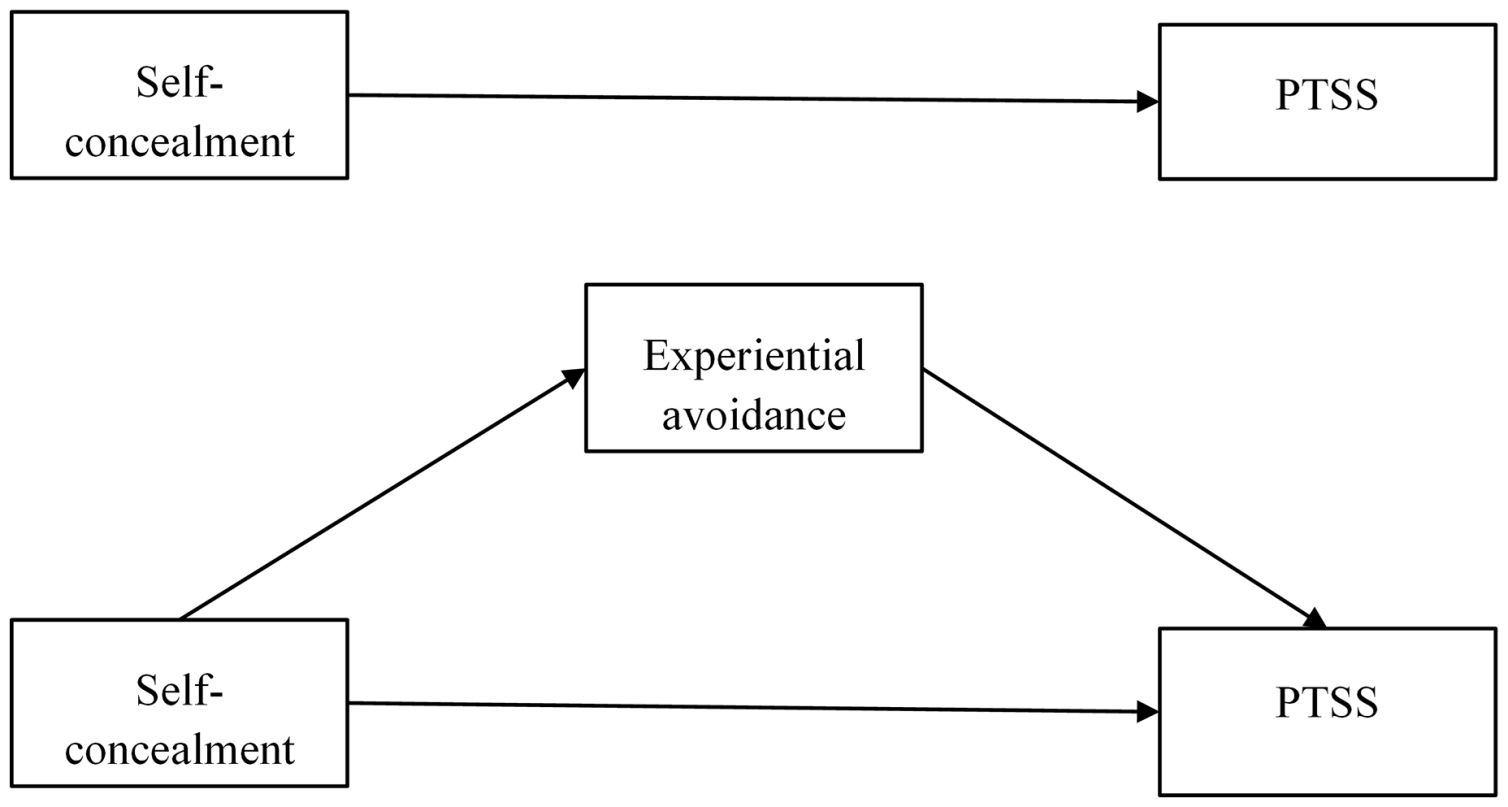
Diagram of the research hypothesis model.

The hypothetical model for this study is shown in Fig 1.

Currently, no studies have examined self-concealment, experiential avoidance, and PTSS in breast cancer patients by integrating them into a unified model Based on the proposed hypotheses, this study aimed to (1) test whether self-concealment significantly predicts PTSS, and (2) test the mediating role of experiential avoidance between self-concealment and PTSS. The results may provide guidance to healthcare professionals in helping patients develop emotion management strategies to alleviate PTSS and promote mental health.

## Materials and methods

### Participants

A cross-sectional design was used in this study. From 15/08/2021 to 31/12/2021, breast cancer patients were recruited as study subjects in the oncology department of a tertiary hospital in Deyang City. This study was conducted from August to December 2021 using a convenience sampling method, selecting breast cancer patients who were rechecked in outpatient clinics or wards of the oncology department of a tertiary hospital in Deyang City. Inclusion criteria were as follows: ① Met the diagnostic criteria for primary breast cancer in the Chinese Anti-Cancer Association Breast Cancer Diagnostic and Treatment Guidelines and Criteria (2019 edition) [24] and was diagnosed with primary breast cancer. ② Knew about the condition for more than 1 month and was within 1 year of completing treatment such as surgery, chemotherapy, or radiation. ③ Aged ≥18 years. ④ No other serious stressful events have been experienced within 1 year;⑤ Participated voluntarily. Exclusion criteria included: ① Metastatic tumor lesions or other malignant tumors. ② Mental or cognitive disorders. Lactation or pregnancy.

**Fig 2:**
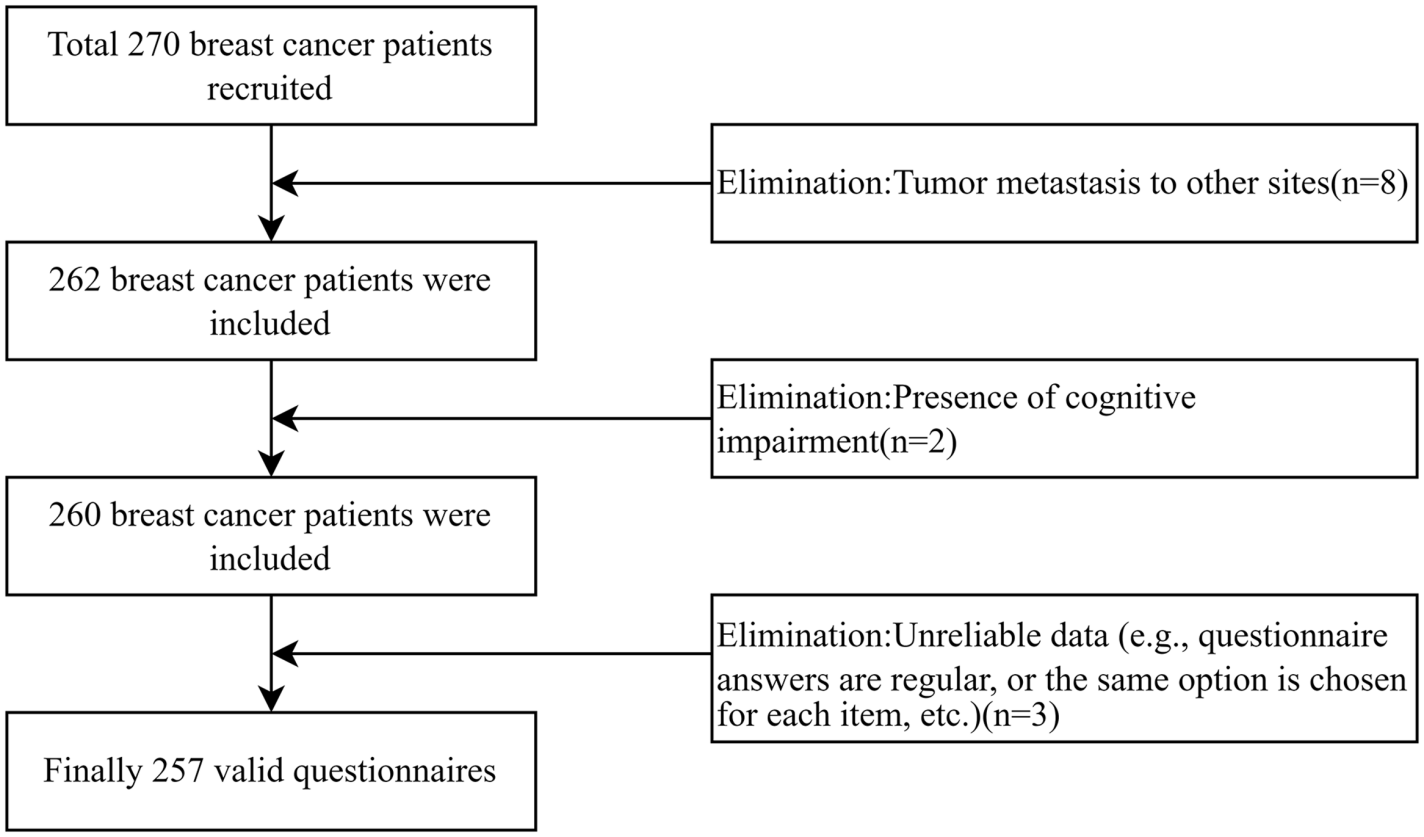
The process of participant selection.

The sample size was calculated using the formula n = [u_α_*σ/δ]^2^ for cross-sectional surveys, with α set at 0.05, uα = 1.96, tolerance error δ = 2.0, and previous studies showing σ = 15.41 [25]. This resulted in a calculated sample size of 193 cases. Considering a 20% dropout rate, a sample of 232 cases was needed. A total of 270 breast cancer patients were recruited to fill out the questionnaire in this study. 8 breast cancer patients were excluded due to tumor metastasis to other sites, 2 breast cancer patients were excluded due to the presence of cognitive impairment, 3 questionnaires were excluded due to unreliable data (e.g., questionnaire answers were regular, or the same options were selected for each item, etc.), and 257 valid questionnaires were examined, with a valid recovery rate of 95.2%. Fig 2 showed the process of participant selection.

### Measures

#### The general information questionnaire.

There were eight entries, including age, number of children, marital status, educational level, religious affiliation, occupational status, average monthly family income, and clinical stage.

### Impact of event scale- revised(IES-R)

This questionnaire was developed by Horowitz [26] and revised by Weiss [27] to assess PTSS in people who have experienced a serious life event. The scale includes three dimensions: intrusiveness, avoidance, and hypervigilance, with a total of 22 items. It is scored on a 5-point Likert scale ranging from 0 (asymptomatic) to 4 (extremely severe), with a total score ranging from 0 to 88. Higher scores indicate more severe PTSS. The Cronbach’s alpha coefficients for the dimensions of the scale range from 0.83 to 0.89. The Chinese version was adapted by Huang Guoping et al [28]., and the Cronbach’s alpha coefficient was 0.96. In this study, the Cronbach’s alpha coefficient for the IES-R was 0.931.

### Self-concealment scale(SCS)

The questionnaire was developed by Larson [29] and the Chinese version was adapted and revised by Wang [30] to assess patients’ tendency to self-conceal. The Cronbach’s alpha coefficient was 0.87. The scale consists of 10 items scored on a 5-point Likert scale ranging from 1 (very nonconforming) to 5 (very conforming), with a total score ranging from 10 to 50. Higher scores indicate a greater tendency toward self-concealment. In this study, the Cronbach’s alpha coefficient for the SCS was 0.794.

### Acceptance and action questionnaire-second edition(AAQ-II)

This questionnaire was developed by Bond [31]and the Chinese version was revised by Cao [32] to assess patients’ experiential avoidance. The Cronbach’s alpha coefficient was 0.92. The scale consists of 7 items scored on a 7-point Likert scale ranging from 1 (never true) to 7 (always true), with a total score ranging from 7 to 49. Higher scores indicate a higher degree of experiential avoidance. In this study, the Cronbach’s alpha coefficient for the AAQ-II was 0.756.

### Data collection

Before the survey, the investigator conducted training and assessments for all personnel involved in the study. This training included the inclusion and exclusion criteria of the research subjects, the purpose and content of the study, the use of the scale, and the precautions for the survey. During the survey, the investigator explained the purpose, content, and precautions for filling out the survey to the patients. Written informed consent was obtained from the respondents via the first page of the paper form, they filled out the survey anonymously on a one-on-one basis. For patients who had difficulty completing the questionnaires due to low literacy levels or old age, the investigator recorded their oral answers. The questionnaires were collected on the spot, and the investigator checked for omissions and errors. After data collection, the data were double-checked by two people using Epidata 3.1 for double-entry, and a third person verified the accuracy. The data underlying this research can be found in the S1 Data.

### Statistical methods

Statistical analyses were performed using SPSS 26.0. Q-Q plots and histograms were used to determine that the data were approximately normally distributed. Measures were expressed as means and standard deviations, and counts as frequencies and percentages. Independent samples t-tests were used to compare PTSS scores across demographic groups (children, religious affiliation). One-way analysis of variance (ANOVA) was used to compare PTSS scores across demographic groups (age, marital status, education level, occupational status, average monthly family income, and clinical stage). Harman’s single factor test was used to check for common method bias. Pearson’s correlation analysis was used to explore the relationships between self-concealment, experiential avoidance, and PTSS. Process 3.5 plug-in was used to test for mediating effects of stratified regression. Bias-corrected percentile Bootstrap was used to test for the mediating effect of experiential avoidance in the relationship between self-concealment and PTSS in breast cancer patients. Significance was assessed by a two-tailed test, and the significance level was set at *P* < 0.05.

### Ethical considerations

The study was reviewed by the Ethics Committee of Deyang People’s Hospital (Ethics Approval Number: 202104052K01). Written informed consent was obtained from the respondents via the first page of the paper form. All collected data were protected and kept confidential in accordance with national and hospital data privacy policies.

## Results

### Characteristics of the breast cancer patients and the differences in PTSS scores

This study included 257 female patients with breast cancer, aged 29–73 years (47.49 ± 8.14 years). The age distribution was as follows: 20–39 years old, 39 cases (15.2%); 40–59 years old, 202 cases (78.6%); and ≥60 years old, 16 cases (6.2%). Among them, 239 cases (93.0%) had children, and 215 cases (83.7%) were married or cohabiting. The educational levels were: illiteracy, 12 cases (4.7%); elementary school, 38 cases (14.8%); junior high school, 111 cases (43.2%); senior high school, 37 cases (14.4%); and college and above, 59 cases (22.9%). Additionally, 47 cases (18.3%) had religious affiliations. Regarding occupational status, 66 cases (25.7%) were employed, 48 cases (18.7%) were retired, and 143 cases (55.6%) were unemployed. The average monthly family income was < 5000 RMB for 174 cases (67.7%), 5000–9999 RMB for 45 cases (17.5%), 10000–19999 RMB for 14 cases (5.5%), and ≥20000 RMB for 24 cases (9.3%). Clinically, 124 cases (48.2%) were in stage I, 91 cases (35.4%) in stage II, 32 cases (12.5%) in stage III, and 10 cases (3.9%) in stage IV. The differences in post-traumatic stress symptom scores among breast cancer patients were statistically significant (p < 0.05) based on demographic factors such as age, children, religious affiliation, occupational status, and average monthly family income (Table 1).

**Table 1:** Characteristics of the breast cancer patients and the differences in PTSS scores.

Projects	Cases	Constituent ratio (%)	Mean ±SD	t/F	*P*
**Age (yr)**					
**20-39**	39	15.2	33.87 ± 14.02	10.01^a^	＜0.001
**40-59**	202	78.6	30.86 ± 13.93		
**≥60**	16	6.2	46.50 ± 8.12		
**Children**					
**Yes**	239	93.0	32.77 ± 14.49	4.52^b^	＜0.001
**No**	18	7.0	25.89 ± 5.10		
**Marital status**					
**Unmarried**	2	0.8	22.50 ± 2.12	3.57^a^	0.076
**Married/cohabiting**	215	83.7	31.43 ± 14.17		
**Divorced/separated/widowed**	40	15.5	37.42 ± 13.24		
**Educational level**					
**Illiteracy**	12	4.7	30.83 ± 6.52	2.02^a^	0.093
**Elementary school**	38	14.8	30.71 ± 18.83		
**Junior high school**	111	43.2	33.56 ± 13.56		
**Senior high school**	37	14.4	36.19 ± 14.64		
**College and above**	59	22.9	28.78 ± 11.78		
**Religious affiliation**					
**Yes**	47	18.3	28.23 ± 10.74	−2.66^b^	0.009
**No**	210	81.7	33.20 ± 14.67		
**Occupational status**					
**Employed**	66	25.7	34.29 ± 13.52	0.99^a^	0.014
**Retired**	48	18.7	32.44 ± 15.83		
**Unemployed**	143	55.6	31.32 ± 13.83		
**Average monthly family income(Yuan)**					
**＜5000**	174	67.7	32.09 ± 14.89	4.99^a^	0.002
**5000-9999**	45	17.5	37.76 ± 11.68		
**10000-19999**	14	5.5	22.79 ± 3.77		
**≥20000**	24	9.3	29.08 ± 12.87		
**Clinical stage**					
**Stage Ⅰ**	124	48.2	33.86 ± 15.94	1.27^a^	0.286
**Stage Ⅱ**	91	35.4	30.20 ± 12.86		
**Stage Ⅲ**	32	12.5	31.56 ± 11.20		
**Stage Ⅳ**	10	3.9	34.20 ± 14.14		

a H-value;

b t-value.

### Common method biases test

To reduce possible common method bias due to self-reported data collection, this study used Harman’s single-factor test. The explained variance of the unrotated first factor was 29.08%, which is less than the critical value of 40%. Additionally, more than one factor had eigenvalues greater than 1, indicating that there is no serious common method bias in this study.

### Descriptive and bivariate statistics

**Fig 3:**
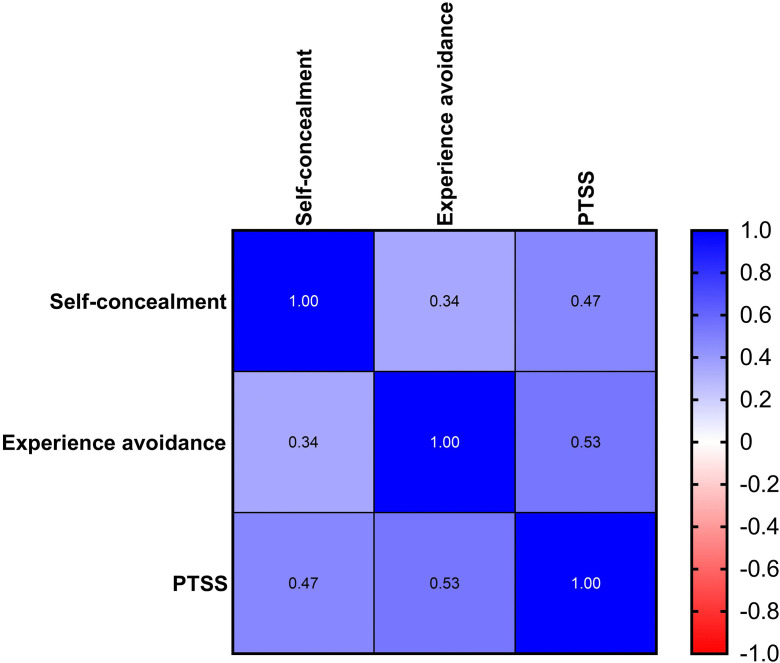
Spearman correlation heat map. ^*^Darker colors indicate stronger correlations between the two indicators.

Breast cancer patients had self-concealment scores of 24.75 ± 7.34, experiential avoidance scores of 18.48 ± 5.44, and post-traumatic stress symptom scores of 32.29 ± 14.14. Correlation analyses showed a positive correlation between self-concealment and experiential avoidance (r = 0.343, *P* < 0.01), and between self-concealment and PTSS (r = 0.467, *P* < 0.01). Experiential avoidance was also positively correlated with PTSS (r = 0.534, *P* < 0.01). The results are summarized in Table 2 and Fig 3.

**Table 2:** Descriptive and correlations analysis for study variables.

Variable	Mean±SD	Rating range	Pearson R correlation		
			1	2	3
**1. Self-concealment**	24.75 ± 7.34	10-50	1		
**2. Experience avoidance**	18.48 ± 5.44	7−49	0.343^**^	1	
**3.PTSS**	32.29 ± 14.14	0-88	0.467^**^	0.534^**^	1

** *P* < 0.01.

### Simple mediation analysis

Multiple linear regression analysis was performed using model 4 of the Process 3.5 plug-in, following the multiple mediation effects validation method proposed by Fang [33]. The differences in total PTSS scores among breast cancer patients were statistically significant in terms of age, children, religious affiliation, occupational status, and average monthly family income (Table 1). Therefore, these variables were set as control variables. Furthermore, the regression coefficients of the control variables represent the unique contribution of that variable to the dependent variable after controlling for other variables. The sign and magnitude of these coefficients should be understood in the context of the specific model. This study mainly focuses on the relationship between the independent variable and the mediating variable. The results are as follows: Equation 1: Self-concealment in breast cancer patients positively predicts PTSS. Equation 2: Self-concealment in breast cancer patients positively predicts experiential avoidance. Equation 3: Experiential avoidance mediates the positive predictive effect of self-concealment on PTSS (Table 3).

**Table 3:** Linear regression analysis results.

Variable		Equation 1 PTSS			Equation 2 Experience avoidance			Equation 3 PTSS		
		*β*	*SE*	*t*	*β*	*SE*	*t*	*β*	*SE*	*t*
**Control variable**	**Age**	0.233	0.127	1.836	−0.359	0.134	−2.678^**^	0.388	0.115	3.386^**^
	**Children**	−0.216	0.221	−0.976	−0.513	0.234	−2.195^*^	0.006	0.199	0.031
	**Religious affiliation**	0.312	0.142	2.201^*^	0.273	0.150	1.819	0.194	0.127	1.526
	**Occupational status**	−0.194	0.067	−2.900^*^	−0.073	0.071	−1.032	−0.162	0.060	−2.719^**^
	**Average monthly family income**	−0.083	0.059	−1.405	−0.068	0.063	−1.088	−0.054	0.053	−1.016
**Independent variable**	**Self-concealment**	0.453	0.055	8.229^**^	0.329	0.058	5.655^**^	0.310	0.052	5.963^**^
**Intermediary variable**	**Experience avoidance**							0.432	0.053	8.112^**^
	**R** ^**2**^	0.516			0.421			0.648		
	**Adj.R** ^**2**^	0.266			0.177			0.419		
	***F***	15.087			8.977			25.686		
	***P***	＜0.001			＜0.001			＜0.001		

* *P* ＜ 0.05.

** *P* ＜ 0.01.

**Fig 4:**
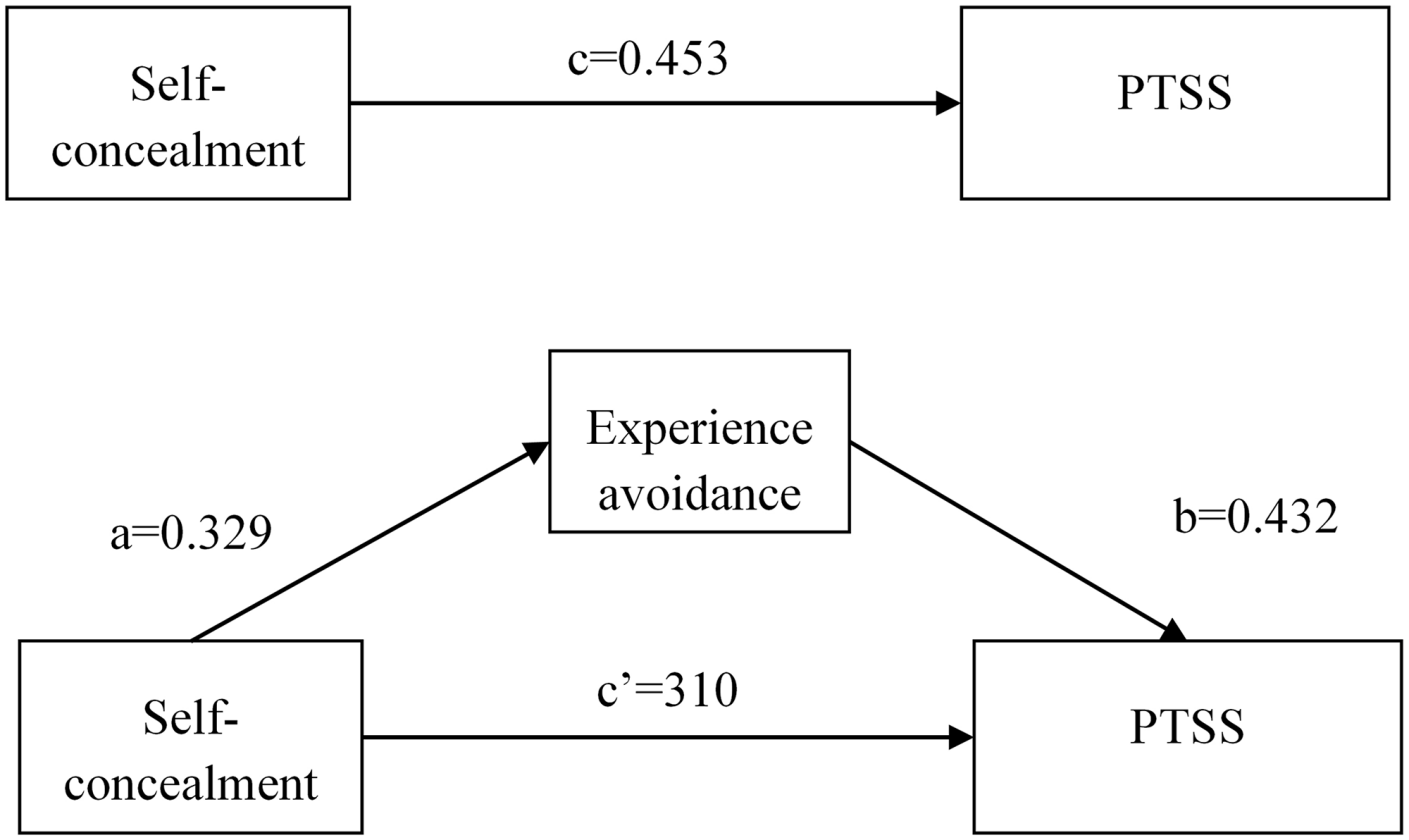
Mediating effect of experiential avoidance between self-concealment and PTSS.

The mediating effect of experiential avoidance between self-concealment and PTSS in breast cancer patients was examined using the bias-corrected nonparametric percentile Bootstrap method with 5000 repetitions and 95% confidence intervals. Without accounting for experiential avoidance as a mediator, the total effect estimate indicated that self-concealment was significantly and positively associated with PTSS (H1: β = 0.453, SE = 0.055, 95%CI [0.344 ~ 0.561],*P* < 0.001). When experiential avoidance was included in the model as a mediator, path analysis estimates showed that self-concealment significantly and positively predicted experiential avoidance (H2: β = 0.329, SE = 0.058, 95%CI [0.215 ~ 0.444],*P* < 0.001), while experiential avoidance significantly and positively predicted PTSS (H3: β = 0.432, SE = 0.053, 95%CI [0.327 ~ 0.537],*P* < 0.001). The mediation analysis indicated that self-concealment has a significant positive indirect effect on PTSS through the pathway of increased experiential avoidance (H4 [indirect effect]: β = 0.142, SE = 0.038, 95%CI [0.074 ~ 0.223], *P* < 0.001). The resulting direct effect estimate was significantly positive (β = 0.310, SE = 0.052, 95%CI [0.208 ~ 0.413], *P* < 0.001), suggesting partial mediation. Specifically, experiential avoidance mediated 31.35% of the relationship between self-concealment and PTSS (Table 4, Fig 4).

**Table 4:** Mediation analysis results for the indirect effect of self-concealment on PTSS through the pathway of experience avoidance.

Hypotheses	Estimates (95% CI)		
	Total effect	Direct effect	Indirect effect
**H1:Self-concealment→PTSS**	0.453 (0.344 ~ 0.561)	0.310 (0.208 ~ 0.413)	
**H2:Self-concealment→Experience avoidance**		0.329 (0.215 ~ 0.444)	
**H3:Experience avoidance→PTSS**		0.432 (0.327 ~ 0.537)	
**H4:Self-concealment→Experience avoidance→PTSS**			0.142(0.074 ~ 0.223)

All path coefficient was standardized.

## Discussion

### Current status of post-traumatic stress symptoms in breast cancer patients

The total score of PTSS in breast cancer patients in this study was 32.29 ± 14.14, which is considered moderate according to the scoring criteria [34] and higher than the scores reported in Li’s study on breast cancer inpatients [25]. This difference may be attributed to the fact that the patients in this study had completed treatments such as surgery, chemotherapy, or radiotherapy within the past year. The short duration since diagnosis and treatment may have heightened their fear of cancer recurrence and progression, triggering avoidance behaviors to escape the psychological stress and pain caused by the disease. Consequently, their PTSS remained at a high level. Additionally, the various examinations and treatments during follow-up visits repeatedly exposed the patients to traumatic events, hindering their cognitive processing of these experiences. This difficulty in processing trauma made it hard for them to perceive and express their inner thoughts and emotions. As a result, they became more reluctant to confront cancer and its related events, inducing or exacerbating PTSS.

### The effect of self-concealment on post-traumatic stress symptoms in breast cancer patients

Mediation effects analyses in this study showed that, without accounting for experiential avoidance as a mediator, self-concealment was significantly and positively associated with PTSS (H1: β = 0.453, *P* < 0.001). When experiential avoidance was included as a mediator, self-concealment still had a significantly positive direct effect on PTSS (β = 0.310, *P* < 0.001), supporting hypothesis H1. Research indicates that self-concealment is a natural and evasive defense mechanism that often occurs after exposure to a life-threatening traumatic event [10]. Breast cancer patients, who experience changes in appearance and increased psychological stress due to cancer, surgery, and radiotherapy, often hide or repress their painful feelings and experiences as a means of self-protection [35,36]. However, self-concealment does not alleviate psychological pain but rather causes its accumulation, exacerbating psychological issues such as anxiety, depression, and PTSS [37,38]. The Sexual Minority Stress Model suggests that self-concealment is linked to recent psychological stress from traumatic events. Individuals who hide information constantly monitor and inhibit disclosure, leading to cognitive resource depletion and increased psychological stress and negative emotions [14]. Additionally, a tendency toward self-concealment hinders the development of adaptive coping strategies. Individuals with higher self-concealment tendencies are less likely to seek external support, delaying the resolution of PTSS. The Dual-Motive Conflict Model supports this view, stating that internal conflicts from the opposing impulses to withhold and disclose information deplete self-regulatory resources. This depletion leads to physical discomfort, mental health problems, and poor social relationships [10]. Therefore, a higher tendency toward self-concealment is associated with higher levels of PTSS in breast cancer patients. For breast cancer patients, reducing self-concealment extends beyond merely alleviating feelings of shame; it also serves to prevent further depletion of psychological resources. Clinically, efforts should be focused on identifying the sources of patients’ stigma and establishing a non-judgmental communication environment. This approach can facilitate a transition from a state of “self-protection” to one of “seeking social support,” thereby disrupting the vicious cycle of PTSS.

### Mediating effects of experience avoidance in breast cancer patients between self-concealment and post-traumatic stress symptoms

Mediation effect analyses in this study revealed that self-concealment significantly predicted experiential avoidance (H2: β = 0.329, *P* < 0.001), and experiential avoidance significantly predicted PTSS (H3: β = 0.432, *P* < 0.001). There was a mediating effect of experiential avoidance between self-concealment and PTSS (H4: β = 0.142, SE = 0.038, 95% CI: 0.074–0.223, *P* < 0.001), accounting for 31.35% of the total effect. These findings support hypotheses H2, H3, and H4. The Self-Concealment Working Model states that high levels of self-concealment motivation drive various goal-directed behaviors (e.g., secrecy, behavioral avoidance, lying) and emotion-regulating strategies (e.g., expressive inhibition) used to conceal negative or distressing personal information [10]. Thus, the more pronounced an individual’s tendency to self-conceal, the higher their level of experiential avoidance. Specifically, when individuals are inclined to conceal negative information, they actively engage in secrecy behaviors, thereby avoiding the disclosure of their painful experiences to others [39]. This secrecy represents a form of experiential avoidance. Moreover, behavioral avoidance, which distances individuals from situations and topics related to negative information, serves to further exacerbate the degree of experiential avoidance. Studies have noted that while trauma survivors’ use of experiential avoidance strategies to escape from trauma-related events can temporarily alleviate PTSS, the persistent avoidance of psychologically distressing stimuli can further increase PTSS levels [21]. From an emotional processing perspective, experiential avoidance leads to difficulties in emotion regulation, hindering the effective management of negative emotions and exacerbating PTSS in patients [40]. Therefore, the higher the degree of experiential avoidance in breast cancer patients, the more severe their PTSS. Conversely, a lower degree of experiential avoidance can help alleviate PTSS in these patients.

The Self-Concealment Working Model states that self-concealment contributes to mental health problems, including anxiety, depression, and PTSS, primarily through increased secrecy behaviors and dysfunctional emotion regulation processes [10]. Individuals with a higher propensity for self-concealment tend to use dysfunctional emotion regulation strategies, exacerbating PTSS. Conversely, those with a lower propensity for self-concealment are more likely to use positive emotion regulation strategies, alleviating PTSS. Specifically, when breast cancer patients learn of their diagnosis, those with a high tendency for self-concealment are more inclined to adopt experiential avoidance to repress their pain. They avoid scenes and memories related to their cancer diagnosis and treatment as a form of self-protection. In the short term, this avoidance may help patients temporarily escape the psychological impact of the traumatic events and reduce their PTSS. However, continuous avoidance leads to a rebound effect, keeping patients in a state of high alertness for extended periods. As they are repeatedly intruded upon by traumatic memories, they passively and continuously recall the pain associated with these events, resulting in the aggravation of PTSS.

### Practical implications

Our research, grounded in the Self-Concealment Working Model and the Dual Representation Theory of post-traumatic stress responses, focuses on breast cancer patients. It explores how personality traits of self-concealment and emotional regulation strategies of experiential avoidance influence post-traumatic stress responses in these patients. This deepens our understanding of such responses and encourages medical staff to pay more attention to them. It also aids in quickly identifying, screening, and assessing patients’ stress responses, setting the stage for effective interventions.

For psychological intervention, medical staff can use cognitive-behavioral therapy to help patients recognize and alter negative thought patterns, reducing self-concealment tendencies. They can also conduct acceptance and commitment therapy to encourage patients to gradually confront trauma – related memories, situations, and emotions, lowering experiential avoidance. Additionally, eye movement desensitization and reprocessing (EMDR) can be used to promote the reintegration of traumatic memories through eye movements and other bilateral stimuli, alleviating PTSS.

In terms of social support, medical staff should urge patients to build good social relationships with family, friends, and fellow patients, creating a platform for sharing and support. Regular peer – exchange activities can be organized, and professional training can be provided for family members on disease knowledge and psychological support skills to boost their ability to care for patients. Furthermore, professional psychotherapists or counselors can lead psychological support groups, organizing regular activities for patients to anonymously share inner feelings and reducing the psychological pressure from self-concealment.

In personalized intervention, a multi – disciplinary medical team should be formed, including oncologists, psychotherapists, nurses, and rehabilitation therapists. Based on patients’ self-concealment and experiential avoidance levels, as well as their personality traits, personalized intervention plans should be developed. These plans should combine various intervention methods to enhance the effectiveness of interventions.

### Limitations

The present study has several limitations. First, due to the research team’s resource constraints and the exploratory nature of the research question, we employed a cross – sectional survey to examine the mediating role of experiential avoidance between self – concealment and post – traumatic stress responses in breast cancer patients. As this design involves single – time – point data collection, it cannot effectively establish the temporal sequence of variables, thus limiting causal inference. Second, This study recruited breast cancer patients from only one hospital, so it is unknown whether the findings can be generalized to other populations. Third, self-reported data were used to assess self-concealment, experiential avoidance, and PTSS, which may introduce bias. Finally, it is important to note that beyond experiential avoidance, other potential mediating variables, such as ruminative thinking and cognitive appraisal, as well as potential moderating variables, may also play significant roles in influencing the relationship between self-concealment and PTSS in breast cancer patients. However, these variables were not examined in the current study. This limitation may restrict the comprehensiveness of the findings and hinder a full elucidation of the complex underlying mechanisms at play. Future research could adopt a prospective cohort design with stratified sampling to select participants from diverse regions and hospitals, ensuring better population representation. Extended follow – up periods with multi – time – point surveys would help establish temporal sequences and dynamic interactions between variables like self – concealment, experiential avoidance, and post – traumatic stress responses. Additionally, expanding the study scope to include other cancer groups, disease populations, or even individuals with psychological disorders or healthy cohorts would enrich our understanding of the psychological mechanisms at play. Multi – center, large – scale studies could further enhance the generalizability of findings. Ultimately, future research endeavors should integrate variables such as ruminative thinking, cognitive reappraisal, and other potential mediators and moderators to develop a more robust and comprehensive model elucidating the intricate psychological mechanisms at play.

## Conclusion

In summary, this study further explored the relationship between self-concealment, experiential avoidance, and PTSS in breast cancer patients. Building on theories such as the Self-Concealment Working Model and Dual Representation Theory of PTSS, the results showed that self-concealment can directly and positively predict PTSS in breast cancer patients. Additionally, self-concealment indirectly affects PTSS through experiential avoidance. These findings are significant for alleviating PTSS and promoting the psychological health of breast cancer patients.

## Supporting information

S1 Data De-identified study data. (XLSX)
